# “It is a thing that depends on God”: barriers to delaying first birth and pursuing alternative futures among newly married adolescent girls in Niger

**DOI:** 10.1186/s12978-019-0757-y

**Published:** 2019-07-18

**Authors:** Ghazaleh Samandari, Carolyn Grant, Lily Brent, Sara Gullo

**Affiliations:** 1 School of Public Health, University of North Carolina at Chapel Hill, Chapel Hill, USA; 2 151 Rue Faubourg St. Antoine, 75011 Paris, France; 3 CARE USA, 151 Ellis Street NE, Atlanta, GA 30303 USA

## Abstract

**Background:**

Pregnancy among adolescent girls in Niger contributes to 34% of all deaths among females ages 15–19, but there is a dearth of research as to the specific contextual causes. In Zinder region, an area that is especially impoverished and where girls are at heightened risk, there is very little information on the main obstacles to improving adolescents’ health and well-being. This qualitative study examines the underlying social, individual and structural factors influencing married girls’ early first birth and participation in alternative opportunities (such as education or economic pursuits) in Niger.

**Methodology:**

In July of 2017, researchers conducted in-depth interviews with a non-probability sample of community members in three communes of Zinder Region, Niger. Participants (*n* = 107) included adolescent girls, husbands of adolescent girls, influential adults, community leaders, health providers, and positive deviants. All interviews were transcribed, coded and analyzed using Dedoose software.

**Results:**

Participants recognize the health benefits of delaying first birth, but stigma around infertility and contraceptive use, desire for children, and belief that childbirth is “God’s will” interfere with a girl’s ability to delay. Girls’ social isolation, lack of mobility or autonomy, and inability to envision alternatives to early motherhood compound the issue. Participants favor adolescents’ pursuit of increased economic opportunities or education, but would not support delaying birth to do so.

**Conclusions:**

Findings indicate the need for a holistic approach to delaying early birth and stimulating girls’ participation in economic and educational pursuits. Potential interventions include mitigating barriers to reproductive health care; training adolescent girls on viable economic activities; and providing educational opportunities for girls. Effective programs should also include or target immediate members of the girls’ families (husbands, parents, in-laws), influential local leaders and members of the community at large.

## Resume Français

**Contexte:**

La grossesse chez les adolescentes au Niger représente 34% de tous les décès chez les femmes âgées de 15 à 19 ans, mais les recherches sur les causes contextuelles spécifiques sont rares. Dans la région de Zinder, région particulièrement appauvrie, où les filles courent un risque accru, très peu d’informations existent sur les principaux obstacles à l’amélioration de la santé et du bien-être des adolescentes. Cette étude qualitative examine les facteurs sociaux, individuels et structurels sous-jacents qui influencent la première naissance précoce des filles mariées et leur participation aux possibilités alternatives (telles que l’éducation ou les activités économiques) au Niger.

**Méthodologie:**

En juillet 2017, les chercheurs ont mené des entretiens approfondis avec un échantillon non probable de membres de la communauté dans trois communes de la région de Zinder, au Niger. Les participants (*n* = 107) comprenaient des adolescentes, des maris d’adolescentes, des adultes influents, des leaders communautaires, des prestataires de soins de santé et des déviants positifs. Toutes les interviews ont été transcrites, codées et analysées à l’aide du logiciel Dedoose.

**Résultats:**

Les participantes reconnaissent les avantages pour la santé de retarder la première naissance, mais la stigmatisation entourant l’infertilité et l’utilisation de contraceptif, le désir d’avoir des enfants et la conviction que l’accouchement est. “la volonté de Dieu” interfèrent avec la capacité de la fille de retarder. L’isolement social des filles, leur manque de mobilité ou d’autonomie et leur incapacité à envisager des alternatives à la maternité précoce aggravent la situation. Les participants sont favorables à la recherche par les adolescents de meilleures opportunités économiques ou d’éducation, mais ne soutiendraient pas le report de la naissance.

**Conclusions:**

Les résultats indiquent la nécessité d’une approche globale pour retarder la naissance précoce et stimuler la participation des filles aux activités économiques et éducatives. Les interventions potentielles comprennent la réduction des obstacles aux soins de santé en matière de reproduction; former des adolescentes à des activités économiques viables; et offrir des possibilités d’éducation aux filles. Des programmes efficaces devraient également inclure ou cibler les membres immédiats de la famille des filles (mari, parents, beaux-parents), les dirigeants locaux influents et les membres de la communauté en général.

## Plain English summary

Rapid pregnancy among newly married adolescents in Niger is a major cause of death among young girls in the country. Especially in poor, rural areas such as the Zinder region of the country, these girls have limited access to resources that would allow them to prevent pregnancy or to pursue any life opportunities beyond motherhood. CARE International sought to understand the social, cultural and structural reasons why girls continue to get pregnant at such early ages, in order to design a targeted intervention to help girls pursue better futures. CARE researchers carried out in-depth interviews with 107 people in this region including adolescent girls, husbands of adolescent girls, family members, community leaders and health providers. Respondents recognized the health and economic benefits of delaying first birth among young girls. However, newly married couples feel pressure from their communities to get pregnant right away and many individuals believe that pregnancy timing is up to “God’s will”. Moreover, girls in this region are isolated, have low education and lack access to health care and economic opportunities, making it difficult for them to envision or pursue a more prosperous life. The findings from this study suggest that a multi-faceted intervention is needed which includes training adolescent girls in education or economic activities, reducing barriers to reproductive health care and increasing overall community support for delaying rapid first birth among newly married girls.

## Resume en Français simple

La grossesse rapide chez les adolescentes nouvellement mariées au Niger est. une cause majeure de décès chez les jeunes filles du pays. Dans les zones rurales pauvres telles que la région de Zinder, ces filles ont un accès limité aux ressources qui leur permettrait d’empêcher une grossesse ou de poursuivre toutes les opportunités de la vie au-delà de la maternité. CARE International s’est. efforcée de comprendre les raisons sociales, culturelles et structurelles pour lesquelles les filles continuent de tomber enceintes à un âge aussi précoce, afin de concevoir une intervention ciblée visant à les aider à améliorer leur avenir. Les chercheurs de CARE ont mené des entretiens approfondis avec 107 personnes dans cette région, notamment des adolescentes, des maris d’adolescentes, des membres de leur famille, des dirigeants communautaires et des prestataires de soins de santé. Les répondants ont reconnu les avantages pour la santé et l’économie de retarder la première naissance des jeunes filles. Cependant, les couples nouvellement mariés ressentent la pression de leurs communautés pour tomber enceinte tout de suite et beaucoup de personnes pensent que le moment de la grossesse dépend de la “volonté de Dieu”. De plus, les filles de cette région sont isolées, ont un faible niveau d’éducation et n’ont pas accès aux soins de santé ni aux opportunités économiques, ce qui les empêche d’envisager ou de mener une vie plus prospère. Les résultats de cette étude suggèrent qu’une intervention à multiples facettes comprenant la formation des filles à l’éducation ou aux activités économiques, la réduction des obstacles aux soins de santé en matière de procréation et l’augmentation du soutien de la communauté pour retarder la première naissance rapide chez les filles nouvellement mariées.

## Introduction

Niger has among the poorest sexual and reproductive health outcomes of any country in the world, including the highest fertility rate (7.6 children per woman) and a maternal mortality rate of 590 deaths per 100,000 births [1, 2]. Adolescent girls in this context are at significant risk; the average age of first marriage is 15.7 years and over 70% of adolescents are married by the age of 19. The adolescent birth rate in Niger is 207 per 1000 women ages 15–19 years; and even among the youngest adolescents, 12.8% of girls have already given birth before the age of 15 [3, 4]. Furthermore, maternal mortality among adolescents accounts for 34% of all deaths in this group [2].

A host of factors drive early childbirth among adolescents. Family planning among young married adolescents can be impeded by inadequate knowledge of contraceptives and partner dynamics which limit their individual ability to make decisions about preventing early pregnancy [5–7]. Family, community and entrenched social norms for gender roles, which tie a girl’s value to her ability to procreate, also yield powerful influence over young couples’ fertility choices [8, 9]. Finally, adolescents experience an inordinate number of obstacles to accessing reproductive services within the formal health system, including bias of providers, stigma against contraceptive use, and lack of physical or financial access to health facilities. [10–13].

In Zinder region, where adolescent girls face a host of barriers to sexual and reproductive health and choice, over 52% of adult women experience their first pregnancy by age 19. Average fertility in Zinder is 8.5 children and knowledge of modern methods of contraception is lower than the country average (79.5% Zinder vs. 89.3% overall) [2]. Zinder is also a religiously conservative area where prohibitions on contraceptive use set forth by spiritual leaders is even more pronounced [14]. Extreme poverty and lack of basic health services in this region has been further compounded in recent years by massive in-migration due to the threat of the militant Islamist group, Boko Haram, which has spilled over the border into southeastern Niger. While Zinder itself has so far been shielded from direct attacks, there have been reports of increased sequestering of young women and girls from public spaces and further limitations in their mobility due to the threat of attack [15].

The current evidence base on the reproductive health and lives of adolescent married girls in Niger is sparse. Particularly in the Zinder region, there is little research on the most important drivers of early birth among married adolescents and potential strategies to support girls in delaying birth. The purpose of this research is to understand the obstacles to and supports for delaying early first birth among adolescent girls in the Zinder region of Niger. Furthermore, we aim to investigate the barriers that married adolescent girls face when opting to pursue alternatives opportunities (such as education or employment) in lieu of immediate childbirth. The findings from this formative research study can be used to inform development of interventions targeted towards improving the health and well-being of adolescent girls in this context.

## Methodology

### Study design and setting

In July of 2017, researchers conducted in-depth interviews with a non-probability sample of community members in three districts of Zinder Region, Niger: Dogo, Droum and Koleram. These sites were selected in consultation with the CARE Niger team and local stakeholders on the basis of both accessibility and suitability for intervention. The CARE team also targeted areas of Zinder where other partners were not already implementing programs aimed at delaying childbirth among adolescent girls (although other sexual and reproductive health or adolescent programs may exist).

### Study subjects and sampling

Researchers targeted a non-probability sample of adolescent girls under the age of 20 years (married *n* = 21, unmarried *n* = 20) as well as husbands of adolescent girls (*n* = 21), influential adults (*n* = 15), community leaders (*n* = 15), health providers, (*n* = 13), and positive deviants (married adolescents who purposefully delayed birth for at least 2 years, *n* = 2). Both married and unmarried girls were included to understand the continuum of support needed for young adolescents as they transition from their natal homes into life as a married woman. Health providers included both physicians, nurses and midwives based at facilities as well as community health workers operating within target villages.

Within each district, local data collectors, trained by CARE staff, asked community leaders to assist them in identifying married and unmarried adolescents for participation in interviews. Adolescents, in turn, referred their husbands or influential adults in their lives for recruitment. Health care providers from local health centers and communities were also interviewed. Written or oral (depending on literacy) informed consent was collected from all participants prior to their interview. This study was reviewed and received ethical approval by the Niger Ministry of Public Health. Adolescents were recruited on the basis of these recommendations, and given informed consent procedures directly (if they were married and considered emancipated) or given assent procedures and asked for parental consent (if they were single and under the age of 18). Only a very small proportion of the candidates we approached refused participation.

### Data collection and storage

Interviewers from the local population who were fluent in the local language were selected to minimize bias and increase comfort among participants. Female and male participants were also matched to interviewers of the corresponding gender. All interviews were held in a private area within the village and audio-recorded with the consent of participants, and each participant was interviewed individually. Each interview involved two data collectors: one conducting the interview using a pre-tested semi-structured interview guide, and one taking notes. At the end of each data collection day, recordings and notes were collated by the research team leader and transported to the CARE Niger office for storage in a locked cabinet. Only relevant members of the research team and program staff had access to these materials throughout the course of the study. No individual names or identifiers of participants were recorded.

### Data analysis

Data from the interviews were transcribed and translated from Hausa into French and analyzed for content. Analysis involved coding the data, developing a list of emerging themes, categorizing the themes within a hierarchical framework of main and sub-themes, using a grounded theory approach. A sample of interviews were double-coded for inter-rater reliability and quality assurance purposes. Where there were discrepancies or disagreements with coding or interpretation, the data analysis team reflected on the text together to determine meaning. All coding was done using Dedoose software.^1^

## Results

### Demographic background

Table 1 describes the demographic characteristics of participants. The average age of unmarried girls was 14 years, while married girls averaged approximately 16 years of age. Literacy was very low among all participants and none of the adolescents reported being employed or in school. The average age of husbands interviewed was 22.5 years, and nearly half were employed or engaged in income-generating activities. Typical jobs included commerce, farming and services such as pest control. Influential adults included mothers and grandmothers, as well as a few in-laws, one father and one grandfather. Community leaders consisted mainly of imams and village elders. Of the 13 health providers interviewed, only 6 reported receiving specific training in family planning.

**Table 1:** Basic demographic characteristics of participants

Participant type	Average age (years)	% reporting literacy	% attended any school	% with engaged in income-generating activity	% Muslim
Adolescent girls (unmarried, *n* = 20)	14	30% (*n* = 6)	30% (*n* = 6)	0% (*n* = 0)	100% (*n* = 20)
Adolescent girls (married, *n* = 21)	16	20% (*n* = 5)	20% (*n* = 5)	0% (*n* = 0)	100% (*n* = 20)
Husbands (*n* = 21)	22.5	33% (*n* = 7)	14% (*n* = 3)	47% (*n* = 10)	100% (*n* = 21)
Influential adults (*n* = 15)	38	7% (*n* = 1)	7% (*n* = 1)	27% (*n* = 4)	100% (*n* = 15)
Community leaders (*n* = 15)	45	20% (*n* = 3)	7% (*n* = 1)	20% (*n* = 3)	100% (*n* = 15)
	% with FP training	% Physician	% Nurse	% CHW	% agree FP best after first birth
Providers (*n* = 13)	43% (*n* = 6)	7% (*n* = 1)	43% (*n* = 6)	50% (*n* = 7)	79% (*n* = 11)

### Advantages to delaying birth

Respondents reported several benefits to delaying childbirth, including the adolescent’s health and well-being, as well as the well-being of existing or future children. In particular, there was acknowledgment that some married adolescents’ bodies are not physically mature enough to safely carry and bear children.

*Her husband will not have a problem since his wife will not have problems with childbirth. The family members will also have no problems because they will then think that she has become mature. Thus, she will not have problems with childbirth or a vaginal tear. The family will not have to worry about the problems she may have in childbirth or about her future.* – female community leader

Some respondents, notably the adolescent girls themselves, expressed understanding of or interest in benefits such as income-earning opportunities or pursuit of education if an adolescent she delayed the birth of her first child. Some respondents noted that these benefits may extend beyond the adolescent girl, to her husband and parents.

*When I'm married I will opt for birth spacing and study during this time. Because children prevent a woman from studying.* – unmarried adolescent

*A young woman who does not have a child has many advantages, for example, can move freely, go to the field, find cooking wood and even visit people. The parents can benefit from the help of this woman if she does business. The most important benefit is to her husband.* – married adolescent with children

Some of the older, female participants also emphasized the importance of delaying pregnancy until the girl has reached physical maturity and even suggested that delaying pregnancy for 2 years after marriage is ideal.

*The marriage can be done at the age of 14 or 15 but the first pregnancy does not occur until 17 years. The advantage is to be mature before giving birth. Young people can be up to two years before childbirth, but it all depends on the age at which the daughter is married, the younger she is, the longer the pregnancy takes. –* female community leader

*Some delay for two years. This is really the ideal age to avoid the risks of complications during childbirth. From a health point of view, they mostly face problems during childbirth, some require assistance to give birth and even caesarean delivery for others. If they are study[ing], having a child will hamper their studies. They cannot do anything to make money.* – influential female

### Obstacles to delaying childbirth

Participants named a number of obstacles to delaying childbirth among newly married adolescents, mainly stigma and reprisal from family and community members. Adolescents who choose to delay birth can be perceived as infertile or disobedient. The specter of infertility can bring shame upon the girl as well as her family, and she can be rejected by her husband or ostracized by the community.

*Two years without a child? They will think that she is sick, they will say that she must be treated so that she can get pregnant.* – married adolescent w/o child

*People will consider her a fool because how a woman can stay up to 2 years without giving birth? Here the brides give birth at the first chance. She may also be considered as a barren woman, a woman who cannot bear a child. The other parents will say they should give birth each year and that their daughter made the exception so we must look for medicines [for her]. The other members of the community will say that she is barren and will laugh at her. –* unmarried adolescent

Another key obstacle to delaying birth is the risk of being seen as going against God’s will. In these communities, pregnancy and childbirth are viewed both as part of one’s divine duty, as well as an event that is left to God’s choice. Interfering with the process of conception and birth can be interpreted as rebuking one’s religious duty, putting the couple at additional risk of stigma from the community.

*They will see [the adolescent] as one who is mad because not following the divine prescriptions. They will see the husband as someone who is mad for not following the divine prescriptions … The parents will be frowned upon because they did not intervene in the face of the couple's decision.* – unmarried adolescent

*Among my relatives, no one is going to support me in order to delay the birth because for them it goes against the religious prescriptions* – married adolescent with children

*How can I support delaying pregnancy when it is a thing that depends on God*? – influential female

Although knowledge of modern methods was high among adolescent participants, there is strong stigma against nulliparous married adolescents using any methods to delay their first pregnancy. For many participants, contraceptives are seen as a way to space birth, but not to delay first birth. Particularly for a newly married adolescent, the idea of using contraceptives to delay is judged negatively by community members.

I think it's not normal for a childless married teenager to use contraceptives. For those with first or second children, they can use them to give their children the chance to grow in the best conditions*.*

*–*influential female

People in the community will take you for crazy. Looks like she's [mentally] sick. Spacing or delay is only allowed after the first pregnancy*.*

*–* married adolescent without children

Even health providers are subject to the same community and social norms associated with contraceptive use, sometimes exhibiting bias against delaying birth or method use among adolescents. Of the health care providers interviewed, several noted that they would not recommend or prescribe contraceptive methods for married adolescent girls who had not yet had their first birth.

*Yes, she can delay her pregnancy and take contraceptive methods when she has her first child.* – physician

*No, me I will not give her, how could a teenage girl who is married take products?* – community health worker

*Providers will advise her to wait until she has her first child before considering contraceptives. If the bride has a child, it is not a problem in that she seeks to space births.* – nurse

In addition to general stigma against contraceptive use, there are some myths and misconceptions about contraceptive products that complicate delaying birth. Some, including health providers, believe that using contraceptive methods prior to the first birth may cause infertility or future miscarriages.

If she asked me permission I would not let her do it unless she did so without my knowledge, and if she did, I would divorce her. For me, why is she going to take a product that will make her sick, which can make her sterile? Any product that will deprive you of pregnancy for three years will surely prevent her from being pregnant her whole life*.*

*–* husband of adolescent w/ children

For me [using contraceptives before first birth] is not good because if you start with contraceptive products it could harm her procreation*.*

– community health worker

While delaying birth was largely condemned in this context, the concept of spacing births *after* the arrival of the first child had more appeal than deferring the initial pregnancy. Respondents noted that spacing births can benefit the well-being of the woman, child and family overall. Contraceptive method use is also seen as an appropriate means to space birth, but not to prevent or delay the first birth.

*People will disapprove because she never gave birth and she tries to delay [first birth]. It is after a first pregnancy that one normally tries to delay the following pregnancy* – married adolescent without children

*When you have too many children, you will face poverty … For the woman, the spacing or the delay of the births will allow her to always be in a good state of health. Otherwise, if she does not, she will get tired quickly. For the husband, the advantage lies in the fact that his money will not be depleted.* – husband without children

*No there is no problem [using contraception] as long as it is for spacing birth and not for delaying birth.* – husband with children

### Advantages to pursuing economic/educational opportunities

Participants were asked to relay the potential benefits that could stem from an adolescent girl pursuing other activities while she delayed motherhood. Her potential economic contribution to the family was seen as desirable for both her husband and her parents and in-laws.

She would be happy, even the members of her family would be happy. Because she continued her studies to the point of having a job and helping her family members*.*

– husband of adolescent with children

*I can delay my first birth so that I can achieve my goals (studies). This will allow me to help my parents depending on what I earn.* –unmarried adolescent

Participation in education or income-generating activities could also be a means of increasing adolescent girls’ autonomy. Respondents cited a girl’s ability to provide for herself and her family as means of being less dependent on the husband, seen as something positive in this case.

With economic activities the earnings will allow her to have an autonomy so that she can have everything she needs without expecting me although I have an obligation to her. And in some measure it can even help me in many things. For her parents or her in-laws, she can help them; for the children she will have in the future, they will have everything they desire. For the community, it can serve as a relief for any needy person*.*

– husband w/o children

*Any economic activity is beneficial. In 1 year or 2 years she finds, she can do her own activities. She can buy livestock for breeding because we will have to breed. The advantage of the husband, by virtue of this activity, she will not frequently ask him for money, and will provide for her wants without the assistance of the husband. Too much request in a couple kills the marriage. She can help her parents too. If a woman works, before the husband satisfies a need for the children, she can do it.* –husband with children

Children were also mentioned as potential beneficiaries of an adolescent girl’s better future:

*I will support it because I have seen its importance and that our children can grow up healthy and have what they want. –* husband without children

### Obstacles to pursuing economic/educational opportunities

The biggest potential obstacle to an adolescent pursuing opportunities is the disapproval of the husband. The husband’s consent is essential for a married adolescent girl to be able to pursue education, skills training or labor participation.

*The most difficult obstacle for her is her husband because if he says no, even if her parents allow it she cannot do otherwise than to follow the husband's decision. –*husband of adolescent with child

*I can have problems if my husband forbids me [to work]. Even if my parents encourage me. If he says no, I will obey his orders.*– married adolescent w/o children

For some participants, delaying birth to pursue work was seen as an inappropriate choice, implying that the adolescent girl’s primary duty is to be a mother and wife. Furthermore, pursuing educational and economic activity is not considered entirely incompatible with childbearing, and earning money is not seen as a good enough reason to go against social and religious norms of immediate childbearing.

No, you don’t have to delay the first pregnancy for any reason. Pregnancy is also an opportunity, why pursue other opportunities if we have one right in front of us. It is unimaginable for a young woman to delay her pregnancy for business, it is not done here*.*

– husband of adolescent with children

[If she delays birth to pursue an alternative opportunity] the community can make gossip and stigmatize it*.*

*–* husband with children

### Support needed to delay birth

Respondents were asked to explain what types of support a married adolescent girl would need in order to be able to delay birth. Three main elements emerged as being essential. First, husbands are seen as having ultimate power of decision-making over the adolescent. The next most important source of support comes from the immediate family, namely parents and in-laws. Finally, participants felt that adolescent girls need access to family planning knowledge and services in order to be able to effectively delay birth.

*Her husband can support her. It is only he who can support it because it is the only one who has power over it.* – influential female

*It is an agreement between the spouses. The husband consulted his wife and they found an agreement. It’s an agreement that we sign. She will confer with her husband; the biggest support is his support.* – male community leader

*[She needs] knowledge about reproductive health because if she has knowledge herself, she can sensitize or encourage her husband. –* health care provider

In some cases, even the support of husband and family is not enough to avoid reproach by the community and individuals may put direct pressure or judgment on the family or husband to discourage their support for delaying birth.

*Even if by chance the villagers learn that the youngster has had the support of his family they will say that they are not thinking correctly. The in-laws of the girl will also be condemned by the villagers. -* unmarried adolescent

## Discussion

This study fills a significant gap in our understanding of the context-specific drivers of early birth among married adolescent girls in Niger. Our findings suggest a myriad of influences that lead to early childbearing among married adolescent girls in this environment. Despite acknowledging the health and economic benefits to delaying birth, girls are pressured by stigma of infertility, barriers to accessing contraceptives and family influence to get pregnant shortly after marriage. The prospect of economic advancement is not so great as to overtake the barriers to delaying first birth.

Numerous other findings echo the results of this study, particularly as relates to the influence of the husband on decision-making, the stigma associated perceived infertility and lack of access to sexual and reproductive health services. A husband’s dominance in fertility decision-making is well established in other similar settings, and is an oft-cited reason for inclusion of husbands in interventions aimed at delaying or spacing births among women [16, 17]. Furthermore, husbands often dictate married girls’ mobility and access to financial resources, both elements important to obtaining contraceptive methods [18]. Provider misconceptions o contraceptives and bias against youth services present formidable obstacles to adolescent reproductive health and must be addressed as part of any intervention geared towards increasing girls’ ability to delay first birth [19, 20].

Among the most salient influences on childbirth among married adolescent girls in Niger is a fatalistic belief in the will of God to dictate the timing of first birth. Even participants who supported delaying birth or acknowledged benefits thereof ultimately put the outcome of a couple’s fertility in divine hands. This reliance on God’s will to determine the timing of birth may discourage the use of contraceptives or other attempts to delay birth. A recent study of family planning acceptability in Niger showed that religion may play a role in fertility decision-making, but that the opinion of husbands and influential leaders in the community ultimately takes precedence when it comes to contraceptive use and limiting or spacing births [21]. Religious leaders interviewed in this study also conceded that there are no specific Islamic scriptures against modern methods and that religious teachings which address maintaining the health of a woman could be used to encourage delayed birth (data not shown), making them potential allies in the effort to delay early pregnancy among young married girls.

The majority of respondents were in favor of providing married adolescent girls with opportunities for advancing their educational or employment outcomes. However, the promise of economic advancement may not be powerful enough to overtake social pressure for rapid first birth following marriage. These findings indicate the need for effective communication campaigns to demonstrate the long-term health and economic well-being of the girl and her family when she is allowed to delay birth and pursue alternative opportunities. Such a campaign would serve not only to directly educate young brides and their husbands on benefits of delaying birth, but could also help dampen the community stigma around delaying birth that so clearly influence couples’ fertility decisions.

Despite the barriers to delaying first birth, there were a number of encouraging findings that present opportunities for successful intervention. Study participants displayed knowledge of and support for birth *spacing* as a way to protect a woman’s health and ensure the well-being of her children and family. The fact that Nigerien communities are familiar with and approving of birth spacing suggests a foundation upon which more learning and acceptance for delaying birth may be built. Religious leaders may also be employed to correct misconceptions around religious edicts on contraceptive use, and to encourage protection of girls’ health through delayed first birth. Many respondents, particularly husbands and girls themselves, were also supportive of providing girls with educational and employment opportunities.

These findings should be viewed in light of the study limitations. Although we sampled a substantial number of different participants in multiple villages, the findings are not generalizable due to the purposive nature of the sampling and total number of participants. Furthermore, these contextual barriers are specific to the Zinder region in which the data collection was focused, and may not apply to other parts of Niger. Finally, due to the highly sensitive topic of sexual and reproductive health in this context, there may be the presence of response bias.

## Conclusion

Findings from this research suggest the need for a holistic approach to both addressing the barriers to pregnancy prevention and stimulating girls’ participation in economic and educational pursuits. While many participants understood the importance of delaying birth for protecting girls’ health and were encouraging of educational and employment opportunities for girls, there was still a great deal of reticence to back the idea of delaying first birth even in order to advance girls’ economic outcomes. These contradictions highlight the complexity of the issue of childbearing in this setting and suggest the need for a multi-faceted approach to intervention which targets socio-behavioral, structural, community level and individual level barriers to delaying rapid first birth among married adolescents.

Promising areas for intervention include addressing mitigating health system barriers to reproductive health care; training adolescent girls on viable economic activities; and providing literacy and educational opportunities for girls. Effective interventions should also include or target immediate members of the girls’ families (husbands, parents, in-laws), influential local leaders and members of the community at large.

## Data Availability

The datasets used and/or analyzed during the current study are available from the corresponding author on reasonable request.

## Footnotes

1 Dedoose is cross-platform online application designed for analyzing a variety of medium for qualitative and mixed methods research. It can be found online at: https://www.dedoose.com/
